# BCMA-CAR-T细胞治疗多发性骨髓瘤后复发与耐药的机制及防治策略

**DOI:** 10.3760/cma.j.issn.0253-2727.2021.09.014

**Published:** 2021-09

**Authors:** 荔 张, 莹 王, 开林 徐

**Affiliations:** 徐州医科大学血液病研究所，徐州医科大学附属医院血液科，江苏省骨髓干细胞重点实验室 221002 Blood Diseases Institute, Xuzhou Medical University; Department of Hematology, the Affiliated Hospital of Xuzhou Medical University; Key Laboratory of Bone Marrow Stem Cell, Xuzhou 221002, China

多发性骨髓瘤（MM）是一种常见的浆细胞血液恶性肿瘤[1]。目前自体干细胞移植（ASCT）、蛋白酶体抑制剂（PI）、免疫调节药物（IMiD）和单克隆抗体（McAb）等治疗提高了MM患者的缓解率和生存率，但大多数患者最终会出现复发或耐药。嵌合抗原受体T细胞（CAR-T细胞）作为一种新的治疗方法，在复发/难治的MM中取得了令人鼓舞的疗效，尤其是以B细胞成熟抗原（BCMA）为靶点的CAR-T细胞[2]–[3]。

BCMA是肿瘤坏死因子超家族蛋白中的一员，主要表达在恶性和正常浆细胞以及一些成熟的B细胞，在重要组织不表达，是治疗MM的有效靶点[4]。国内外临床试验中，以BCMA为靶点的CAR-T细胞治疗复发/难治性MM取得显著疗效，总有效率达73％～100％、完全应答率达31％～69％[5]。尽管BCMA-CAR-T细胞疗法治疗复发/难治MM获得了较高的缓解率，但目前报道出患者的中位无进展生存（PFS）期仅4.1～11.8个月[6]，约45％接受CAR-T治疗的患者在达到缓解之后再度复发[7]。因此复发或耐药是我们目前需要研究探讨的重点问题。本综述主要从CAR-T细胞相关因素、MM细胞自身因素以及肿瘤微环境等方面介绍CAR-T细胞治疗后复发或耐药的相关机制以及应对策略。

一、CAR-T细胞相关因素

研究表明，BCMA-CAR-T细胞治疗后复发的患者大多是BCMA阳性复发，失去了具有活性的CAR-T细胞对MM细胞的监视，即存在CAR-T细胞耗竭和持久性降低。如何有效改善CAR-T细胞在体内的功能及持久性是解决复发或耐药问题的关键。

1. T细胞质量：研究表明，早期谱系细胞，如初始T细胞、干细胞记忆性T细胞越多，T细胞增殖能力越强，对CAR-T细胞发挥疗效至关重要[8]。此外，不同表型的CD4^+^和CD8^+^ T细胞亚群表现出不同的增殖潜力和寿命[9]，CD4^+^/CD8^+^ T细胞比例越高，BCMA-CAR-T细胞在体内扩增能力越强[10]。一项关于BCMA-CAR-T细胞疗法的临床试验中，前期收获的CD27^+^CD45RO^−^CD8^+^ T细胞数量和CAR-T细胞在体内的扩增程度呈正相关[10]。这表明，更有效的BCMA-CAR-T产品可能来自分化较少的初始T细胞和干细胞记忆性T细胞[10]。因此，收集T细胞时选择特定的T细胞表型如初始T细胞、干细胞记忆性T细胞，或制备CAR-T细胞时改变条件使T细胞向特定方向分化[11]，比如在前期T细胞体外扩增时加入IL-7、IL-15细胞因子使其向干细胞记忆性T细胞富集[12]，有助于改善CAR-T细胞的功能，使其发挥更好的肿瘤杀伤作用。

2. CAR结构：CAR共刺激结构域的选择可能会影响细胞的激活、持久和免疫衰竭。和CD28相比，含有4-1BB共刺激结构域的CAR-T细胞体内存活时间更长，并具有更强的增殖能力。研究表明，在体外BCMA+的肿瘤细胞刺激下，以CD28为共刺激分子的CAR-T细胞比4-1BB为共刺激分子的CAR-T细胞凋亡增多。体内实验中，含4-1BB的BCMA-CAR-T细胞比含CD28的BCMA-CAR-T细胞持久性强，更重要的是，4-1BB可以保护BCMA-CAR-T细胞免受BCMA特异性激活诱导的细胞死亡（AICD）[13]。4-1BB还可以促进CAR-T细胞向中枢记忆T细胞（CD45RO^+^CCR7^+^）分化，抑制CAR-T细胞耗竭[14]。此外，优化CAR的结构，如通过限制旁观者免疫细胞Fc受体结合的长IgG4/IgG2衍生间隔区可以增加特异性配体依赖性CAR的激活，关闭配体非依赖式紧张性信号，可能有利于CAR-T细胞的持久性[15]–[17]。

3. 淋巴细胞清除方案：目前常用的是氟达拉滨联合环磷酰胺方案，其疗效相对优于单用环磷酰胺，其机制可能是降低了抗CAR-T细胞免疫反应的风险，使输注的CAR-T细胞在体内有效扩增[1],[15]。Yan等[16]研究发现在复发难治性MM患者中ASCT治疗后再进行CAR-T治疗，CAR-T细胞的扩增比使用相同CAR-T细胞但接受氟达拉滨-环磷酰胺预处理的患者高100倍，表明更强的淋巴清除治疗方案可能促进CAR-T细胞扩增。

4. 增强CAR-T细胞对自然T细胞的诱导：CAR-T细胞诱导的炎症反应可以增强宿主免疫系统识别新抗原，引发天然免疫系统的抗肿瘤反应。天然T细胞通过其细胞受体（TCR）识别其他肿瘤抗原，这一反应可能导致CAR-T细胞靶向抗原阴性的肿瘤细胞使其消除[18]。

二、MM细胞的免疫逃逸

除了CAR-T细胞相关因素外，CAR-T细胞治疗MM后复发或耐药常见的原因之一是肿瘤细胞不再表达靶抗原。部分患者在BCMA-CAR-T细胞治疗后复发或进展时，MM细胞BCMA表达阴性或低表达，克服肿瘤细胞的免疫逃逸对提高CAR-T细胞的疗效至关重要。

1. BCMA的双等位基因缺失：是BCMA-CAR-T治疗MM后抗原阴性逃逸机制之一。Samer等[5]利用深度全外显子测序及单细胞RNA测序技术检测了MM患者接受CAR-T治疗前后的骨髓样本，结果显示大多数MM细胞具有16p13.13的BCMA基因座，16p与17p突变存在于同一克隆中，并且BCMA有一个高亚克隆无意义突变（p.Q38*），该突变在BCMA基因上产生一个早期终止密码子，一个copy的缺失和第二个copy的功能丧失突变可导致BCMA双等位基因缺失，此机制为MM复发时肿瘤细胞表面缺乏BCMA表达提供了分子基础。另一研究通过全基因组测序（WGS）观察到BCMA16号染色体21.3 Mb的杂合缺失和91 kb的纯合缺失（从12 058 001到12 149 000），导致BCMA基因在16p13.13位点的双等位基因丢失，这可能是BCMA-CAR-T治疗后抗原阴性逃逸的另一机制[19]。

针对抗原阴性复发，可以利用靶向多种肿瘤抗原的CAR-T细胞，如不同靶点CAR-T细胞联合或序贯输注，或输注单个有多个独特scFv的bi/trispecific CAR-T细胞以及单个CAR靶向两个抗原的CAR-T细胞，以克服单一靶点CAR-T细胞导致的肿瘤细胞免疫逃逸。Yan等[20]报道了22例接受抗CD19 CAR-T及抗BCMA CAR-T细胞序贯输注，总缓解率95％、严格意义的完全缓解率43％、完全缓解率14％，81％患者微小残留病灶阴性。目前，已构建出抗CD19/BCMA串联CAR-T细胞，小鼠模型中，抗CD19/BCMA串联CAR-T细胞对CD19和（或）BCMA阳性的肿瘤细胞有显著和特异的抗肿瘤作用，抗CD19/BCMA串联CAR-T细胞相对于单一CAR-T细胞来说显示出更好的肿瘤杀伤作用[21]。此外，还有抗BCMA和CS1的双靶点CAR-T细胞[22]，抗BCMA和CD38双靶点CAR-T细胞[23]等正在临床前研究或临床试验中。基于APRIL的第三代CAR-T细胞（ACAR）可以同时靶向BCMA和另一浆细胞相关抗原（TACI）。APRIL是一种紧凑型自身蛋白，可以和MM上的BCMA和TACI抗原高亲和力结合。在小鼠肿瘤逃逸模型中，BCMA^+^TACI^−^和BCMA^−^TACI^+^肿瘤细胞完全被ACAR清除，而只靶向BCMA的CAR-T细胞可导致MM BCMA−肿瘤的生长[24]。探索ACAR的临床试验目前正在进行中（NCT03287804）[25]。无论是两种CAR-T细胞的联合使用还是双靶点CAR-T细胞或同一CAR靶向不同抗原，对复发难治性MM都是新的选择。

2. γ分泌酶复合体的切割：是MM肿瘤细胞靶抗原密度降低的机制之一。多亚基γ分泌酶复合体能够切割MM肿瘤细胞表面的BCMA抗原，不仅降低了肿瘤细胞上的靶抗原密度，还能够释放可溶性BCMA（sBCMA）片段，过多的sBCMA在骨髓中积聚可以抑制CAR-T细胞对肿瘤细胞的识别。目前关于CAR-T细胞疗法和γ分泌酶抑制剂联合治疗复发难治MM的临床试验已启动（NCT03502577），Pont等[21]研究表明，应用γ分泌酶抑制剂可以抑制BCMA从肿瘤细胞表面脱落，使得MM细胞表面BCMA呈剂量依赖式显著增加，同时外周血的sBCMA浓度降低，增强BCMA-CAR-T细胞对MM细胞的识别能力，抗肿瘤效果提升。此外，HDAC7和Sec61抑制剂均可增加MM细胞表面的BCMA表达，和γ分泌酶抑制剂联合使用能更明显增加BCMA表达[26]。

3. 胞啃作用（trogocytosis）：胞啃作用是BCMA-CAR-T细胞治疗MM后肿瘤细胞表面抗原密度降低的另一机制。研究表明，BCMA-CAR-T细胞和KMS-12-BM肿瘤细胞共培养后，肿瘤细胞表面BCMA抗原可逆性降低；相反，CAR-T细胞上的BCMA抗原密度升高。可能机制是胞啃作用将靶抗原转移到CAR-T细胞上，导致CAR-T细胞之间“相互杀伤”，T细胞活性降低并且功能衰竭，当肿瘤细胞表面抗原密度下降到一定程度时，CAR-T细胞则呈无应答状态[17]。可以采取多靶点CAR-T细胞来对抗胞啃作用导致的免疫逃逸问题，减少CAR-T治疗后的复发或耐药。

三、肿瘤微环境

一些BCMA^+^的MM患者虽然在外周血中能检测到BCMA-CAR-T细胞，但仍然出现复发[10]，表明CAR-T细胞的持久性和靶细胞表面抗原的表达可能对于长久的抗肿瘤是必需的，但是还不够充分，比如肿瘤骨髓微环境中普遍存在的免疫抑制因素在复发耐药方面也可能起作用。骨髓微环境中存在各种免疫抑制细胞如骨髓来源的抑制细胞（MDSC）、调节性T细胞（Treg）、调节性B细胞（Breg）等，这些细胞通过免疫抑制、免疫耗竭和免疫抵抗三种主要机制介导MM细胞的免疫逃逸[27]。缓解肿瘤微环境的免疫抑制作用，有望提高CAR-T细胞的疗效。

1. 抑制间充质干细胞（MSC）保护作用：研究表明，在BCMA-CAR-T细胞针对靶细胞具有高亲和力的情况下，MSC对MM细胞无保护作用；相反，当BCMA-CAR-T细胞与靶细胞亲和力较弱时，MSC可以有效保护MM细胞免受CAR-T细胞的杀伤，即CAR-T细胞的亲和力和MSC介导的保护作用之间存在拮抗效果[28]。Survivin、XIAP和Mcl-1抑制剂可以消除MSC这种保护机制，所以MSC介导的免疫抑制可能和抗凋亡蛋白上调有关。我们可以通过增加BCMA-CAR-T细胞的亲和力或者联合应用CAR-T细胞和抗凋亡介质抑制剂来克服MSC细胞在骨髓微环境中的保护作用[28]。

2. 增强T细胞抗肿瘤作用：免疫调节剂（IMiD）对T细胞的有益作用在CAR-T细胞上也已在临床前得到证实[29]，来那度胺可以增强T细胞的功能、改善抑制性骨髓微环境。当来那度胺和BCMA-CAR-T细胞联合应用时，来那度胺以剂量依赖式提高CAR-T细胞的功能，如增强CAR-T细胞分泌细胞因子、溶解细胞，增加外周血中CAR-T细胞的数量，延长存活时间[30]。

3. 免疫检查点抑制剂：PD-1/PD-L1轴是MM细胞最具特征性的免疫检查点通路之一[31]。MM细胞表达的PD-L1和T细胞以及NK细胞表达的PD-1结合，抑制T细胞增殖，降低细胞毒作用和细胞因子的产生，使T细胞功能衰竭。阻断PD-1和或PD-L1可以阻止它们之间的相互作用，增强T和NK细胞抗MM的细胞毒性[32]。研究表明，复发/难治MM患者应用BCMA-CAR-T细胞治疗后，继续使用PD-1抑制剂，可以增强CAR-T细胞扩增和抗MM效果[33]。

4. 组成性表达CD40L：免疫抑制的MM微环境可以通过CAR-T细胞组成性表达CD40L来克服。CAR-T细胞组成性表达CD40L，可以增加促炎因子TH1的分泌，上调肿瘤细胞表面共刺激分子（CD80和CD86）、黏附分子（CD54、CD58和CD70）、HLA分子（ClassI和HLA-DR）和Fas死亡受体（CD95）的表达，增强CD40^+^肿瘤细胞的免疫原性，诱导树突状细胞成熟并分泌促炎细胞因子IL-12，这一系列机制可以促进T细胞增殖、增强抗原呈递DC功能，并有可能招募内源性抗肿瘤免疫应答，增强CAR-T细胞的抗肿瘤效果[34]。

5. 新型全人“装甲”BCMA CAR-T细胞：BCMA2-TbnegCAR是将两个BCMA特异性全人单链可变片段（scFv）通过2A序列双顺反地共表达阴性TGF-βRⅡ，有望改善当前BCMA-CAR-T细胞治疗的有效性和持久性。在BCMA+MM小鼠肿瘤模型中，与“无装甲”BCMA2 CAR-T相比，BCMA2-TbnegCAR表现出更强的增殖能力和细胞毒性，使小鼠肿瘤负荷更早降低，并且可以抵抗MM肿瘤微环境的TGF-β的免疫抑制作用。这一新型CAR-T细胞有望改善目前BCMA-CAR-T细胞的复发耐药问题[35]。

6. 靶向MM细胞所表达抗原的单克隆抗体：①抗CD38单抗：直接靶向免疫细胞，具有免疫调节作用，可以清除骨髓微环境中CD38^+^的免疫抑制细胞，如Treg、Breg和MDSC等[36]。②抗信号淋巴细胞激活分子家族成员7（SLAMF7）单抗：可以抑制MM细胞与骨髓基质细胞（BMSC）黏附，从而抑制MM细胞生长和活性[37]；还可能通过T细胞或其他免疫细胞来干扰MDSC对抗骨髓瘤免疫反应的抑制[38]。靶向肿瘤细胞表达抗原的单克隆抗体是否可以增强CAR-T疗效、减少复发，有待进一步研究。

除了以上列举的复发耐药因素外，患者输注CAR-T细胞前接受的靶向免疫治疗如CD19单克隆抗体可能会增加CAR-T细胞治疗后免疫逃避的复杂性。有文献报道靶向CD19的博纳吐单抗治疗后出现CD19-免疫逃逸[39]。然而，在输注CAR-T细胞之前避免靶向免疫治疗是否对于CAR-T治疗获得持久的长期反应是必要的还有待确定[11]。但可以肯定的是，患者对CAR-T治疗的应答与之前接受的治疗如化疗或基于CD19的免疫治疗之间没有明确的相关性[40]。

四、总结

解决CMA-CAR-T细胞治疗MM的耐药及复发依然面临的严峻挑战。可以通过提高CAR-T细胞在体内的持久性、抑制MM细胞的免疫逃逸、调节MM患者骨髓微环境、探索新的多靶点CAR组合等途径来减少MM患者的复发，提高BCMA-CAR-T细胞的临床疗效，这对MM患者的治疗具有重要意义。

## References

[1] McLellan AD, Ali Hosseini Rad SM (2019). Chimeric antigen receptor T cell persistence and memory cell formation[J]. Immunol Cell Biol.

[2] Mikkilineni L, Kochenderfer JN (2017). Chimeric antigen receptor T-cell therapies for multiple myeloma[J]. Blood.

[3] 吴 晓菲, 王 雅丹, 胡 豫 (2016). 多发性骨髓瘤的CAR-T细胞治疗现状[J]. 中华血液学杂志.

[4] 钟 梦君, 徐 颖茜, 邢 海燕 (2019). 靶向BCMA的嵌合抗原受体T细胞抗多发性骨髓瘤作用研究[J]. 中华血液学杂志.

[5] Samur MK, Fulciniti M, Aktas Samur A (2021). Biallelic loss of BCMA as a resistance mechanism to CAR T cell therapy in a patient with multiple myeloma[J]. Nat Commun.

[6] Shah N, Chari A, Scott E (2020). B-cell maturation antigen (BCMA) in multiple myeloma: rationale for targeting and current therapeutic approaches[J]. Leukemia.

[7] Gagelmann N, Ayuk F, Atanackovic D (2020). B cell maturation antigen-specific chimeric antigen receptor T cells for relapsed or refractory multiple myeloma: A meta-analysis[J]. Eur J Haematol.

[8] Singh N, Perazzelli J, Grupp SA (2016). Early memory phenotypes drive T cell proliferation in patients with pediatric malignancies[J]. Sci Transl Med.

[9] Gattinoni L, Speiser DE, Lichterfeld M (2017). T memory stem cells in health and disease[J]. Nat Med.

[10] Cohen AD, Garfall AL, Stadtmauer EA (2019). B cell maturation antigen-specific CAR T cells are clinically active in multiple myeloma[J]. J Clin Invest.

[11] Shah NN, Fry TJ (2019). Mechanisms of resistance to CAR T cell therapy[J]. Nat Rev Clin Oncol.

[12] Sampson JH, Choi BD, Sanchez-Perez L (2014). EGFRvIII mCAR-modified T-cell therapy cures mice with established intracerebral glioma and generates host immunity against tumor-antigen loss[J]. Clin Cancer Res.

[13] Lam N, Trinklein ND, Buelow B (2020). Anti-BCMA chimeric antigen receptors with fully human heavy-chain-only antigen recognition domains[J]. Nat Commun.

[14] Kawalekar OU, O'Connor RS, Fraietta JA (2016). Distinct Signaling of Coreceptors Regulates Specific Metabolism Pathways and Impacts Memory Development in CAR T Cells[J]. Immunity.

[15] Neelapu SS (2019). CAR-T efficacy: is conditioning the key?[J]. Blood.

[16] Yan LZ, Shang JJ, Kang LQ (2017). Combined Infusion of CD19 and Bcma-Specific Chimeric Antigen Receptor T Cells for RRMM: Initial Safety and Efficacy Report from a Clinical Pilot Study[J]. Blood.

[17] Hamieh M, Dobrin A, Cabriolu A (2019). CAR T cell trogocytosis and cooperative killing regulate tumour antigen escape[J]. Nature.

[18] Majzner RG, Mackall CL (2019). Clinical lessons learned from the first leg of the CAR T cell journey[J]. Nat Med.

[19] Da Vià MC, Dietrich O, Truger M (2021). Homozygous BCMA gene deletion in response to anti-BCMA CAR T cells in a patient with multiple myeloma[J]. Nat Med.

[20] Yan Z, Cao J, Cheng H (2019). A combination of humanised anti-CD19 and anti-BCMA CAR T cells in patients with relapsed or refractory multiple myeloma: a single-arm, phase 2 trial[J]. Lancet Haematol.

[21] Pont MJ, Hill T, Cole GO (2019). γ-Secretase inhibition increases efficacy of BCMA-specific chimeric antigen receptor T cells in multiple myeloma[J]. Blood.

[22] Chen KH, Wada M, Pinz KG (2018). A compound chimeric antigen receptor strategy for targeting multiple myeloma[J]. Leukemia.

[23] Li C, Mei H, Hu Y (2019). Improved efficacy and safety of a dual-target CAR-T cell therapy targeting BCMA and CD38 for relapsed/refractory multiple myeloma from a phase I study[J]. Eur J Immunol.

[24] Lee L, Draper B, Chaplin N (2018). An APRIL-based chimeric antigen receptor for dual targeting of BCMA and TACI in multiple myeloma[J]. Blood.

[25] Schmidts A, Ormhøj M, Choi BD (2019). Rational design of a trimeric APRIL-based CAR-binding domain enables efficient targeting of multiple myeloma[J]. Blood Adv.

[26] Ramkumar P, Abarientos AB, Tian R (2020). CRISPR-based screens uncover determinants of immunotherapy response in multiple myeloma[J]. Blood Adv.

[27] Holthof LC, Mutis T (2020). Challenges for Immunotherapy in Multiple Myeloma: Bone Marrow Microenvironment-Mediated Immune Suppression and Immune Resistance[J]. Cancers (Basel).

[28] Holthof LC, Van der Horst HJ, Poels R (2019). The Impact and Modulation of Microenvironment-Induced Immune Resistance Against CAR T Cell and Antibody Treatments in Multiple Myeloma[J]. Blood.

[29] Wang X, Walter M, Urak R (2018). Lenalidomide Enhances the Function of CS1 Chimeric Antigen Receptor-Redirected T Cells Against Multiple Myeloma[J]. Clin Cancer Res.

[30] Works M, Soni N, Hauskins C (2019). Anti-B-cell Maturation Antigen Chimeric Antigen Receptor T cell Function against Multiple Myeloma Is Enhanced in the Presence of Lenalidomide[J]. Mol Cancer Ther.

[31] Jelinek T, Paiva B, Hajek R (2018). Update on PD-1/PD-L1 Inhibitors in Multiple Myeloma[J]. Front Immunol.

[32] Görgün G, Samur MK, Cowens KB (2015). Lenalidomide Enhances Immune Checkpoint Blockade-Induced Immune Response in Multiple Myeloma[J]. Clin Cancer Res.

[33] Bernabei L, Garfall AL, Melenhorst JJ (2018). PD-1 Inhibitor Combinations As Salvage Therapy for Relapsed/Refractory Multiple Myeloma (MM) Patients Progressing after Bcma-Directed CAR T Cells[J]. Blood.

[34] Curran KJ, Seinstra BA, Nikhamin Y (2015). Enhancing antitumor efficacy of chimeric antigen receptor T cells through constitutive CD40L expression[J]. Mol Ther.

[35] Alabanza L, Vu B, Wu D (2020). A Fully-Human Armored BCMA CAR Boosts Function of CD4+ CAR-T Cells and Resists TGF-β Suppression in Pre-Clinical Models of Multiple Myeloma[J]. Blood.

[36] Zhang L, Tai YT, Ho M (2017). Regulatory B cell-myeloma cell interaction confers immunosuppression and promotes their survival in the bone marrow milieu[J]. Blood Cancer J.

[37] Tai YT, Dillon M, Song W (2008). Anti-CS1 humanized monoclonal antibody HuLuc63 inhibits myeloma cell adhesion and induces antibody-dependent cellular cytotoxicity in the bone marrow milieu[J]. Blood.

[38] Veillette A, Guo H (2013). CS1, a SLAM family receptor involved in immune regulation, is a therapeutic target in multiple myeloma[J]. Crit Rev Oncol Hematol.

[39] Mejstríková E, Hrusak O, Borowitz MJ (2017). CD19-negative relapse of pediatric B-cell precursor acute lymphoblastic leukemia following blinatumomab treatment[J]. Blood Cancer J.

[40] Song MK, Park BB, Uhm JE (2019). Resistance Mechanisms to CAR T-Cell Therapy and Overcoming Strategy in B-Cell Hematologic Malignancies[J]. Int J Mol Sci.

